# High-Intensity Interval Aerobic Resistance Training to Counteract Low Relative Appendicular Lean Soft Tissue Mass in Middle Age: Study Protocol for a Randomized Controlled Trial

**DOI:** 10.2196/22989

**Published:** 2020-10-16

**Authors:** Lara Vlietstra, Debra L Waters, Lynnette M Jones, Kim Meredith-Jones

**Affiliations:** 1 Department of Medicine Otago Medical School, Dunedin Campus University of Otago Dunedin New Zealand; 2 School of Physiotherapy University of Otago Dunedin New Zealand; 3 School of Physical Education, Sport & Exercise Sciences University of Otago Dunedin New Zealand

**Keywords:** sarcopenia, high-intensity interval training, randomized controlled trial

## Abstract

**Background:**

Sarcopenia is the age-related loss of skeletal muscle mass and function and may exist in early middle age. Previous research in this area has focused on resistance training in older individuals; however, there is a lack of intervention trials in middle-aged adults with low relative appendicular lean soft tissue mass who may be at risk for sarcopenia in older age.

**Objective:**

This randomized controlled trial aims to determine the effects of a high-intensity interval aerobic resistance training intervention on appendicular lean soft tissue mass in middle-aged adults with low relative appendicular lean soft tissue mass.

**Methods:**

We will conduct a 40-week, single-blinded randomized controlled trial in 84 middle-aged adults with low appendicular lean soft tissue mass in the wider Dunedin area, New Zealand. We will randomly allocate participants to receive either a group-based, 20-week high-intensity interval aerobic resistance training intervention program or a single, 60-minute education session on current exercise recommendations. After the first 20 weeks, both groups will be given a 20-week home program. The study will assess primary and secondary outcome measures, including body composition (regional and whole-body lean soft tissue mass, fat mass, percentage body fat, measured by dual x-ray absorptiometry), blood biomarkers (cortisol, creatinine, C-reactive protein, lipid profile, hemoglobin), physical fitness (maximum oxygen consumption, blood pressure), physical activity (accelerometry), physical function (handgrip strength, sit-to-stand, gait speed, quadriceps strength), and self-reported questionnaires (health outcomes, self-efficacy, perceived enjoyment of physical activity, and multifactorial lifestyle), at baseline, 20 weeks, and 40 weeks. Physical function and self-reported questionnaires will also be measured at 10 weeks. We will assess the primary outcome measure, total body lean soft tissue mass, at baseline, 20 weeks, and 40 weeks. Analyses will be performed using intention-to-treat principles, comparing the outcomes resulting from the intervention, using linear mixed models.

**Results:**

We obtained ethical approval for this study from The University of Otago Human Ethics Committee on December 10, 2018. Participant recruitment started on February 11, 2019 and was completed on May 14, 2019. Data collection started on February 25, 2019 and was completed on February 28, 2020. We expect to publish the results in January 2021.

**Conclusions:**

High-intensity interval aerobic resistance training is a time-efficient form of exercise, enabling busy middle-aged adults to meet physical activity recommendations while maximizing training results. The findings can inform the development of future prevention-focused interventions aimed at counteracting the high prevalence of sarcopenia in the aging population.

**Trial Registration:**

Australian New Zealand Clinical Trials Registry (ACTRN12618001778279); https://tinyurl.com/y555z6fz.

**International Registered Report Identifier (IRRID):**

DERR1-10.2196/22989

## Introduction

### Background

Sarcopenia is the age-related loss of skeletal muscle mass and function [1]. Due to the gradual decline in muscle mass with age, the focus of research into this disease thus far has mainly been in those over 60 years of age [1]. However, the progressive loss of skeletal muscle mass and strength begins in middle age, and evidence demonstrates that abnormal body composition is apparent before the age of 50 years [2,3]. If abnormal body composition is already present in early middle age, then implementing lifestyle interventions in younger age groups may mitigate the trajectory to low physical function, frailty, and premature death in later life. This could have important public health implications, as older people with sarcopenia are consistently reported to have lower physical function, overall health, and survival than people with normal body composition [4]. Also, low physical activity is common as people grow older [5], increasing the risk for sarcopenia [6].

Two recently published systematic reviews concluded that exercise therapy, with or without combined dietary interventions or supplementation, can be an effective treatment for older adults with sarcopenia [7,8]. However, Yoshimura et al concluded that more high-quality randomized controlled trials (RCTs) are required to confirm these results [7]. To the best of our knowledge, only 3 trials have been conducted in older adults with low muscle mass. A 10-week resistance training program was effective for maintaining functional strength and increasing muscle mass in men and women aged 70 years with low muscle mass [9]. This intervention included moderate- to high-intensity resistance training, where intensity was measured using the Borg CR10 scale, with participants rating their perceived exertion between 6 and 7 out of 10 [9]. Participants performed 8 exercises with the aim of engaging muscle groups in the whole body [9]. In another study, a 12-week multicomponent exercise program improved physical function in sarcopenic or presarcopenic individuals (≥60 years of age) [10]. The intervention consisted of resistance training, balance, flexibility, and aerobic exercises and participants determined their individual resistance load as 12 to 14 on the Borg scale [10]. Finally, a 6-month home exercise program improved physical function in 52 community-dwelling individuals (≥60 years of age) with low muscle mass or sarcopenia [11]. The home program consisted of a combination of walking (20-30 minutes per day) and lower limb resistance exercises (6× squats, 1-minute single-leg standing and 20× heel raises) [11]. To date, to our knowledge, no interventions have been conducted in middle-aged adults with low relative appendicular lean soft tissue mass.

High-intensity interval training (HIIT) was within the top 3 fitness trends for 2016 and is reported to be safe and well tolerated, with adherence that exceeds steady state training [12]. In addition to better adherence, different forms of HIIT are time efficient and provide aerobic fitness and health benefits similar to or better than traditional steady state training [12]. HIIT workouts are usually short and involve aerobic high-intensity exercises, accumulated through short bursts of activity [13]. These short bouts of activity often last between 1 and 4 minutes with a recovery phase between the bouts [13]. A metareview of 33 systematic reviews, including studies across the lifespan, showed that HIIT improved cardiorespiratory fitness, anthropometric measures, blood glucose and glycemic control, arterial compliance and vascular function, cardiac function, some inflammatory markers, and exercise capacity, and decreased heart rate (HR) and increased muscle mass compared with nonactive controls [14]. Different forms of HIIT have been proven safe in different patient populations, including cardiac rehabilitation patients [15,16] and patients with type 2 diabetes [17], rheumatoid arthritis [18], and cancer [19].

To date, to our knowledge, only a few studies have investigated the effects of HIIT on sarcopenia. A recent study in older sedentary adults demonstrated that a combination of HIIT and increased protein intake resulted in a greater increase in mitochondrial content compared with a nonexercise control group, helping to preserve oxidative capacity and slow the process of sarcopenia [20]. The protocol consisted of 5 intervals of 1 minute of stationary cycling at 85% of maximal load reached during maximum oxygen consumption (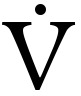
O_2_max) [20]. Another study demonstrated that HIIT can improve skeletal muscle vascularization in older men [21]. The protocol as proposed by Leuchtmann et al consisted of 12 weeks of HIIT followed by 12 weeks of progressive resistance training [21]. The HIIT protocol consisted of seven 1-minute intervals of stationary cycling at 85% of the participants’ peak power [21]. The resistance training consisted of 3 sets of leg extensions, leg press, and squats with a 3-minute rest between sets [21]. After each HIIT and resistance training session, participants received a drink containing 30 g of whey protein [21]. In animal models, HIIT led to a greater muscle mass, larger muscle fiber size, and an increase in mitochondrial biomass in old, sarcopenic, and frail mice compared with nonexercise controls [22]. This research agrees with a study comparing moderate-intensity continuous training versus HIIT in middle-aged rats, showing that HIIT was better at mitigating age-related sarcopenic physiological processes such as oxidative stress and inflammation [23].

The majority of high-intensity training protocols conducted in sarcopenic individuals have been in older adults and are aerobic based. Thus, the opportunity for muscle mass development may be limited. A newer form of high-intensity training using circuit training has recently been proven effective in improving both body composition and strength measurements in middle-aged men and women (50-65 years of age) [12]. In the study by Greenlee et al, 3 center-based trainings a week with at least one day of rest between sessions for 16 weeks, improved muscle mass and muscle strength [12]. The program started with a warm-up and was followed by high-intensity cardioresistance training of 3 sets of 3 to 4 resistance training exercises followed by a set of rope jumping, 4 minutes of high-intensity cardiorespiratory exercises, and 3 sets of 2 to 4 resistance exercises [12]. These exercises were followed by 5 to 15 minutes of whole-body training and 5 to 10 minutes of yoga-inspired flexibility training [12]. HR averaged more than 80% of maximum HR throughout the sessions, and participants spent 66% of the exercise sessions between vigorous and maximal training zones [12].

### Objective

Despite the positive effects of the study by Greenlee et al, to our knowledge, no high-intensity aerobic resistance training (HIART) intervention studies have been conducted in middle-aged adults with low relative lean soft tissue mass. Therefore, the primary aims of this research are to determine whether HIART can increase lean soft tissue mass and whether these changes can be maintained in the long term. Secondary aims include investigating the effect of HIART on biomarkers of sarcopenia, physical fitness, physical activity, physical function, physical activity enjoyment, self-efficacy, and adherence.

## Methods

### Study Design and Setting

The HIIT Your Exercise Target is a single-blinded RCT testing the effectiveness of a 20-week high-intensity interval circuit training intervention on appendicular lean soft tissue mass. We obtained ethical approval for this study from The University of Otago Human Ethics Committee (H18/131) and registered it (ACTRN12618001778279). All participants will provide written informed consent in accordance with the Declaration of Helsinki. This community-based study will be conducted in the Department of Medicine, University of Otago, Dunedin, New Zealand, with training sessions carried out in the Southern District Health Board staff gymnasium, Dunedin.

### Recruitment and Eligibility Criteria

We will recruit sedentary but otherwise healthy participants aged between 40 and 50 years through flyers, community webpage postings, electronic bulletin boards, and local newspapers. Interested people will be directed to complete an online screening questionnaire. Participants will be prescreened for exclusion criteria, exercise safety, and their weekly amount of physical activity. We will deem participants to be eligible to attend a screening appointment if their self-reported physical activity level is below the minimum weekly current exercise recommendations [24]; they do not take medications known to affect body composition or HR; they are not diagnosed with moderate or severe hypertension; they are not pregnant or breastfeeding, or planning on becoming pregnant during the intervention; they are not previously diagnosed with or have symptoms of cardiovascular disease or other serious medical condition; they do not weigh more than 159.9 kg (weight limit of the dual energy x-ray absorptiometry [DXA] scanner); they do not live outside of metropolitan Dunedin; and they are able to communicate in English or te Reo Māori. Further exclusion will occur for exercise safety (see the Exercise Safety Screening subsection below). Participants will receive the information sheet and will be given 2 days to decide if they are willing to participate.

### Exercise Safety Screening

Participants will undergo medical screening, as part of the screening questionnaire, to allow identification of those at high risk of an adverse event during high-intensity exercise. We will do individual medical screening, following the guidelines as proposed by the American College of Sports Medicine/American Heart Association. High-risk participants will be excluded after the online screening questionnaire. All other participants, including medium-risk participants, will be monitored with a 12-lead electrocardiogram (ECG) during the 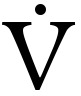
O_2_max test at their first screening appointment. All ECGs will be assessed by an experienced cardiologist and require approval before the participant can enter the study.

### Screening Appointment

Potentially eligible participants will attend 2 appointments as part of the screening process. During the first appointment, a blood sample will be taken, the participant will undergo a total body DXA scan to measure body composition and they will undergo a 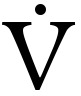
O_2_max test on a stationary bicycle (see outcome measures below for more detail). At the end of the first appointment participants will receive an accelerometer (ActiGraph ) to wear for 7 days and nights to assess physical activity (see outcome measures for more detail). Participants will be emailed a weblink and will be asked to fill out several questionnaires on demographics, generic health outcomes, self-efficacy, physical activity enjoyment, and lifestyle (see outcome measures for more detail).

During the second appointment, 8 days after the first appointment, we will assess gait speed, 30-second sit-to-stand, hand grip strength, and maximal isokinetic and isometric strength of the quadriceps (see outcome measures for more detail). We will apply further exclusion criteria at this point. We will use sex-specific, height squared adjusted cutoff scores as proposed by Prado et al, to classify low appendicular lean soft tissue mass index (ALMI) measured by DXA [25]. Females with an ALMI greater than 7.72 kg/m^2^ and males with an ALMI greater than 9.59 kg/m^2^ will be excluded.

### Blinding, Randomization Methods, and Allocation Concealment

**Figure 1:**
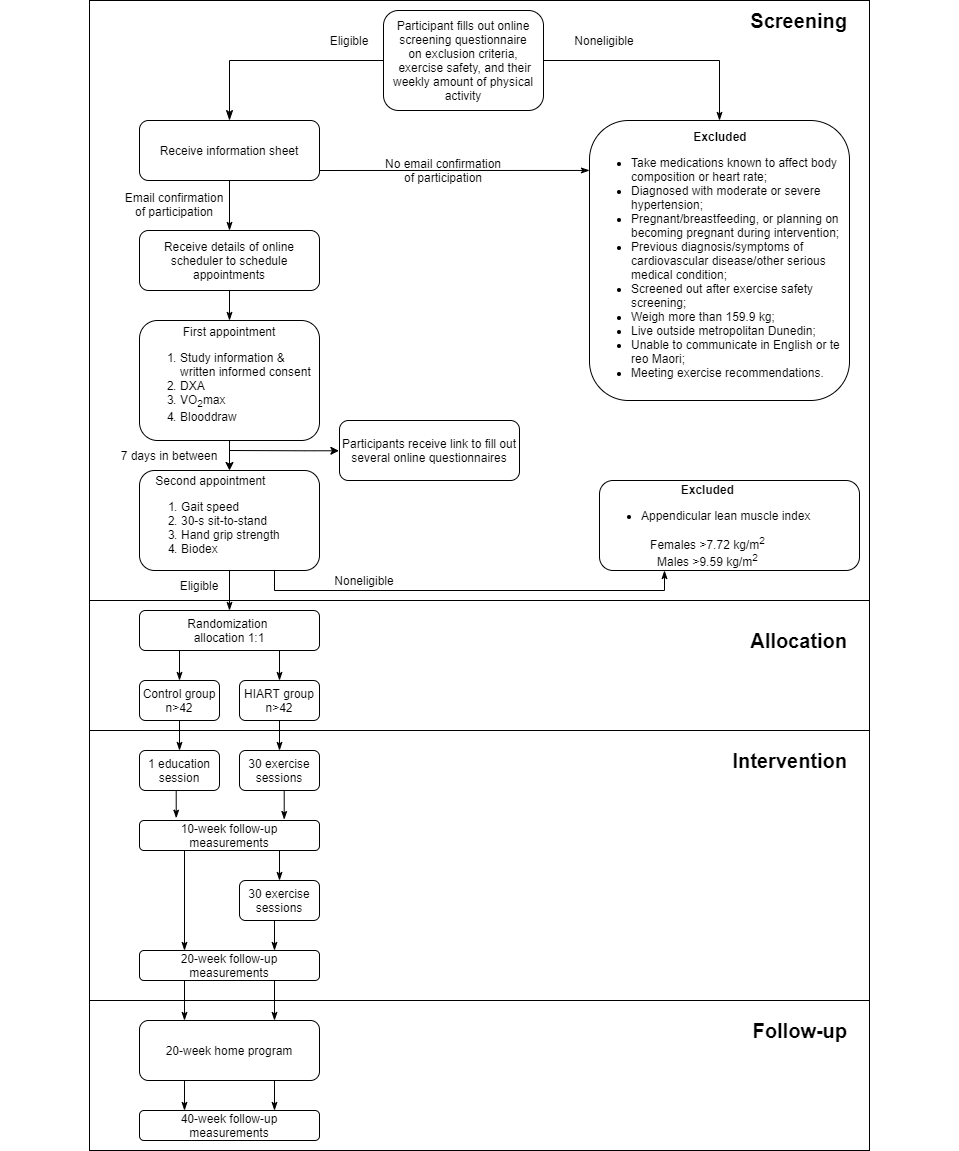
Study design flow diagram. DXA: dual energy x-ray absorptiometry; HIART: high-intensity interval aerobic resistance training; <inline-graphic xlink:href="researchprotocols_v9i10e22989_fig3.png" mimetype="image" xlink:type="simple"/>O_2_max: maximum oxygen consumption.

Figure 1 shows the study flow. Following the screening assessments eligible participants will be randomly allocated to either a control group or HIART group at a 1:1 allocation ratio. Stratified block randomization allocation sequence will be generated in REDCap (REDCap Consortium) and will be used to automatically randomly allocate participants to groups of equal sample size, stratified by sex, age and BMI. The block sizes and allocation sequence will not be disclosed to ensure concealment. The investigator collecting the data will feed data into the computer in separate datasheets, which will be anonymized by a different investigator, to ensure that all other investigators, including lab technicians, cardiologist and the primary investigators remain blinded to the treatment allocation. Statistical analysis will be performed using the anonymized datasets to ensure masking to treatment allocation.

### Intervention

#### Control Group

The control group will receive education on current exercise recommendations during a 60-minute group session, provided by a physiotherapist. We will define current exercise recommendations as proposed by the World Health Organization: adults aged 18 to 64 years should do at least 150 minutes of moderate-intensity aerobic physical activity throughout the week, or at least 75 minutes of vigorous-intensity aerobic physical activity, or an equivalent combination of moderate- and vigorous-intensity activity [24]. Following the 20-week intervention, the control group will be offered 3 weeks of supervised exercise, where they are taught how to perform HIART. After 3 weeks, they will be given access to the remainder of the 20-week home program. We will design the home program specifically for this study and it will be viewable with protected YouTube links.

#### High-Intensity Interval Training Group

The intervention includes a 40-week RCT of HIART. The 40-week intervention involves 20 weeks of training followed by a 20-week follow-up. Each training session will start with a warm-up, followed by HIART, and finishing with a cooldown. HIART involves 3 phases, as follows.

The first is a *power phase* consisting of 2 whole-body exercises, performed for 20 seconds each with maximum intensity, followed by 10 seconds of rest and repeated 8 times. Between each whole-body exercise will be a 1-minute rest. Exercises include jumping jacks, skaters, burpees, and numerous other calisthenics varied by session.

The second is a *cycling phase*, which will be based on the 3-minute all-out protocol as proposed by Gillen et al [26]. We will use stationary spin bikes for this phase (4000GT Spin Bike; AeroSpin). The cycling phase will begin with a 2-minute warm-up, followed by 2 times 4 sets of 20-second all-out sprints interspersed with a 1-minute recovery, followed by a 2-minute cooldown. Participants will be encouraged to increase wattage when appropriate.

Third is a *resistance phase* consisting of 2 resistance exercises with or without free weights. The resistance exercises will target major muscle groups. Exercises will be performed as supersets (antagonistic muscle groups), compound sets (same muscle group[s]), or staggered sets (noncompeting muscle groups; eg, upper and lower body). Participants will perform each exercise 8 times for 20 seconds, followed by a 10-second rest.

Participants will complete all exercises as a circuit with a 1-minute rest between exercises. Training load will be self-selected, but participants will be encouraged to choose a weight or resistance that will ensure that their HR reaches 85% of maximum HR during the high-intensity peaks and does not drop below 60% of maximum HR during the low-intensity periods in the power and cycling phases. All training sessions will be group based, with a maximum of 9 people in each group. Experienced staff with extensive background in group fitness will lead every exercise class. At the end of the 20-week exercise intervention, participants will be given access to the 20-week home program to complete sessions at home.

### Outcome Assessments

**Figure 2:**
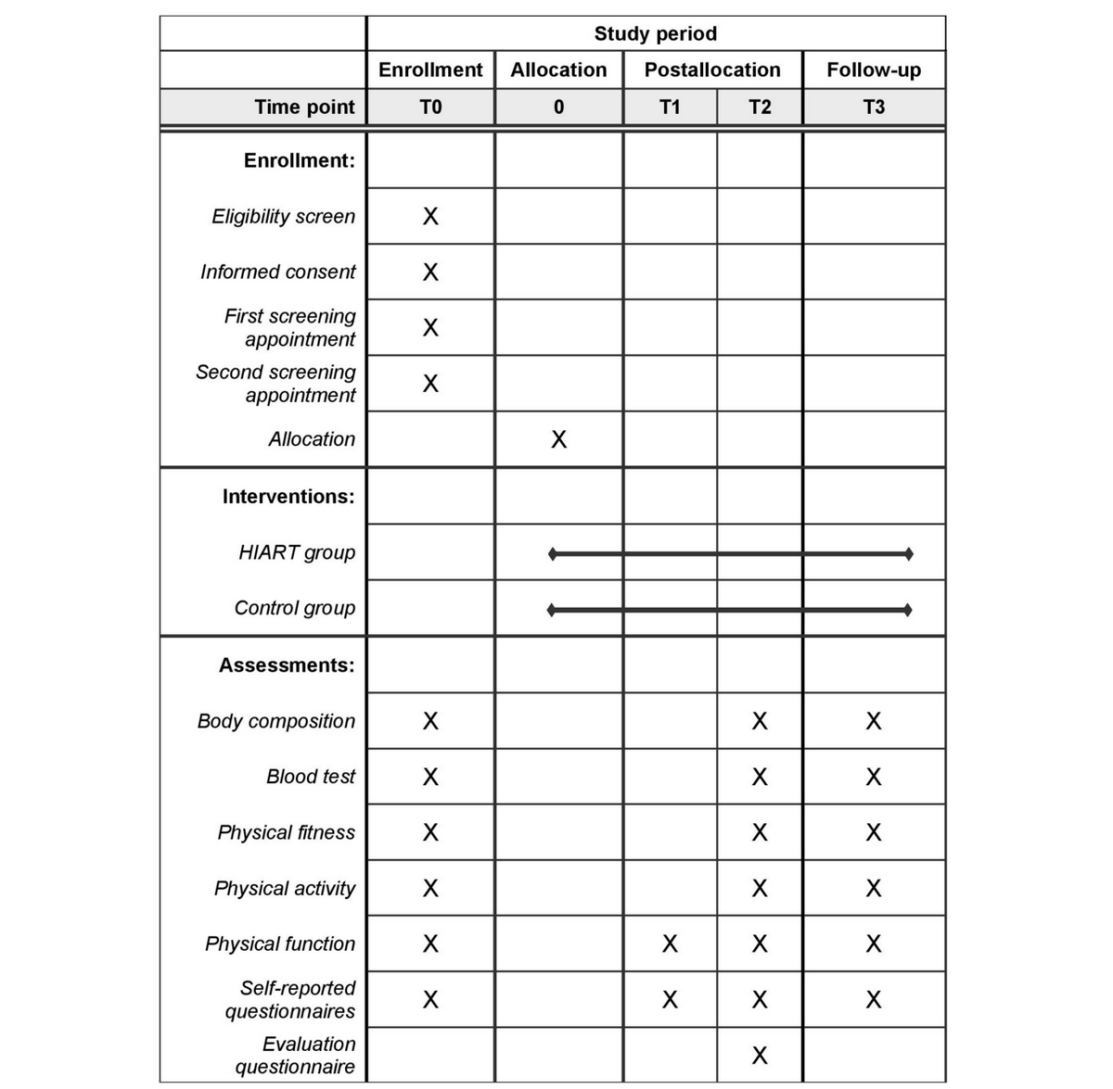
Standard Protocol Items: Recommendations for Interventional Trials (SPIRIT) diagram. HIART: high-intensity interval aerobic resistance training.

We will conduct outcome assessments at T0 (baseline), T1 (midpoint of intervention, 10 weeks), T2 (end of intervention, 20 weeks), and T3 (end of follow-up, 40 weeks) (Figure 2).

### Anthropometry and Body Composition

We will assess body composition by a Lunar Prodigy DXA (Lunar Prodigy; GE Medical Systems) and analyze the results with standard software (Lunar enCORE version 16). The regions of interest for regional body composition will be defined using the software provided by the manufacturer. The scanner will be calibrated daily with phantoms for quality assurance. The laboratory coefficients of variation for repeat in vivo scans in adults in our laboratory are 1.8% for total fat mass, 1.8% for percentage fat, and 1.0% for bone-free lean tissue mass. We will measure height with a fixed stadiometer (Harpenden stadiometer; Holtain, Ltd) and weight with an electronic scale (Seca electronic scale; Seca Corp), both with participants wearing light clothing and with no shoes. Waist circumference will be measured at the top of the iliac crest, upper arm circumference will be measured halfway up the upper arm, and thigh circumference will be measured halfway up the upper leg, with nonelastic tape.

#### Biomarkers

We will measure cortisol, creatinine, C-reactive protein, lipid profile, and hemoglobin by venous blood draw. Blood will be collected at baseline and postintervention. All blood tests will be analyzed by Southern Community Laboratories, Dunedin, New Zealand, using standard procedures. We chose these biomarkers based on previous literature suggesting they are involved in the physiological processes of sarcopenia. A relative increase in cortisol may increase muscle catabolism [27]. Creatinine is a breakdown product of creatine phosphate in muscle and its serum levels are therefore proportional to muscle mass [28]. The literature has demonstrated that inflammatory cytokines (such as C-reactive protein) activate many of the molecular pathways involved in sarcopenia, which could lead to an imbalance between protein synthesis and catabolism [29]. Cholesterol is an essential component of biological membranes and signaling pathways involved in the adaptation of muscle mass to exercise training [30]. Hemoglobin affects the structure and quality of muscle connective tissue through collagen synthesis [31]. Hemoglobin is a marker of nutritional status and has been found to be low in older individuals with sarcopenia [31].

#### Physical Fitness

Participants will perform maximal incremental exercise tests on a cycle ergometer (Model E100 P; COSMED) while blood pressure and metabolic variables (Quark Cardiopulmonary exercise test; COSMED) are monitored and measured. During the first exercise tests we will also measure 12-lead ECG. Resting HR and resting blood pressure will be measured in seated position before the test. Metabolic measures refer to the volume and gas concentrations of inspired and expired air. The protocol will start at 50 W and will be increased at a magnitude of 25 or 50 W (individualized for each participant) every 2 minutes until volitional exhaustion. We will determine the 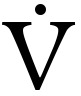
O_2_max, calculate maximum HR as the highest obtained HR, and calculate the HR reserve as the maximum HR minus resting HR.

#### Physical Activity

We will measure physical activity (counts per minute) over 7 days by using an accelerometer attached to a waist strap (GT3X+; ActiGraph). Participants will be asked to wear the accelerometer continuously for 24 hours and physical activity will be analyzed after sleep has been identified and removed.

#### Physical Function

We will measure hand grip strength, gait speed, 30-second sit-to-stand, and quadriceps strength to determine physical function. All physical function measurements will be conducted using standardized encouragement and explanation.

#### Hand Grip Strength

We will measure grip strength using a Saehan model hydraulic hand dynamometer (MSD Europe bvba). The participant will be asked to remove watches, rings, or bracelets and will be given the dynamometer in their dominant hand seated with their back supported by the backrest. Grip strength will be measured with elbows at their side and their elbows at a 90° angle with thumb placed vertically and their feet flat on the floor. Grip strength will be measured in triplicate for each hand, with a 1-minute rest between each test. We will record an average of the 3 trials to determine grip strength.

#### Gait Speed

The participant will be asked to walk down a hallway through a 1-m zone for acceleration, a central 5.5-m testing zone, and a 1-m zone for deceleration. The participant will be asked to walk down the testing zone at normal gait speed and as fast as possible without running, and not to slow down before the 5.5-m mark. Normal gait speed and maximal gait speed will be recorded once.

#### 30-Second Sit-to-Stand Test

We will measure lower extremity strength and endurance with the 30-second sit-to-stand test. The participant will be asked to sit in a standardized chair (a 43-cm high chair, without arm rests, placed against the wall) and asked to sit in the middle, back straight, feet approximately hip width apart and placed flat on the floor, with knees 90° flexed. If needed, one foot can be placed slightly in front of the other to help maintain balance. The participant will be asked to stand and sit as many times as possible in 30 seconds keeping their arms crossed against the chest and instructed to fully sit down between each stand. We will record the number of correct sit-to-stands performed in 30 seconds.

#### Quadriceps Strength

We will measure isokinetic and isometric strength of the quadriceps muscle in both legs using an isokinetic dynamometer (Biodex Corporation). Participants will be seated on the dynamometer with a hip angle of 90° flexion stabilized with thigh and pelvic straps. The chair will be positioned so that the medial condyle of the knee is centered with the axis of the dynamometer with little or no gap behind the knee and the edge of the seat. The lower leg will be secured and the calf pad will be placed 5 cm proximal to the lateral malleolus. The range of motion will be set so as to obtain maximal speed during the isokinetic tests (from 90°of knee flexion to –5° of full knee extension) and at 60° for the isometric tests. Prior to each test, the participants will be given the opportunity to become familiar with the procedures and to warm up, by doing 10 submaximal contractions and 2 maximal contractions. For the test, 6 maximal concentric reciprocal contractions and 3 maximal muscle contractions held for 5 seconds will be completed with a 3-minute rest between.

#### Questionnaires

We will obtain demographic information (age, sex, education, ethnicity, employment, income) at baseline using the relevant New Zealand census questions. To measure generic health outcomes from the participant’s perspective, we will use the 12-item Short-Form Health Survey (SF-12) [32]. To measure exercise self-efficacy, we will use the 18-item Exercise Self-Efficacy Scale (ESES) [33]. We will use the 18-item Physical Activity Enjoyment Scale (PACES) to measure perceived enjoyment of physical activity [34]. To assess lifestyle from a multifactorial perspective, we will use the Lifestyle Appraisal Questionnaire (LAQ) [35]. After the 20-week intervention period, we will measure satisfaction and obtain feedback information from the participants by asking the participants, anonymously, to fill out an evaluation questionnaire.

### Termination Criteria

In the event of chest discomfort, failure of HR to increase normally with increased workload, light-headedness, severe fatigue, and shortness of breath, which are all abnormal responses to exercise, we will advise the participant to discontinue that exercise session. If symptoms continue for longer than 24 hours, the participant will be referred to their general practitioner. If symptoms are recurrent or take longer than 24 hours to resolve, the participant will be withdrawn from the study.

### Adherence

Strategies to improve adherence will include group-based participation [36], individualized exercise goals, flexibility in rescheduling to another session online, and the use of reminders. We will monitor participation adherence using attendance checklists. Adherence to the set HR goals will be monitored by recording the participants’ HR throughout the exercise sessions using HR monitors (Polar RC3; Polar Electro Oy). Adherence to the home program will be monitored with a self-reported physical activity diary.

### Sample Size

Based on a standard deviation of 1.9 kg and an effect size *f* of 0.39 (obtained from previous literature [37]), our study has 95% power using a 2-sided 5% level of significance to detect clinically meaningful differences in lean soft tissue mass of 1.5 kg between the intervention and the control group with 35 participants per group. We will aim to recruit 84 participants (42 per group), which allows for 15% dropout or unusable data.

### Statistical Analysis

All analysis will be conducted in Stata version 15 (StataCorp LP) using the principles of intention-to-treat analysis. We will use descriptive statistics to characterize the groups at baseline. The intention-to-treat analysis for this study will include all participants, including those who are not fully compliant and those with missing outcome data. The primary outcome will be the change in total body lean soft tissue mass. Secondary outcomes include changes in physical fitness, muscle strength, physical function, and blood biomarkers. The primary analysis will compare the primary and secondary outcomes resulting from the intervention, using linear mixed models to model outcomes at T1, T2, and T3 adjusted for baseline values. Standard mixed-model diagnostics will be performed. Group differences will be presented in the form of mean differences for continuous outcomes and an odds ratio for binary outcomes, with their associated 95% confidence intervals.

## Results

We obtained ethical approval for this study from The University of Otago Human Ethics Committee on December 10, 2018. The project is supported by a University of Otago Research Grant and a Dunedin School of Medicine Dean’s Bequest Fund (January 2019 to March 2020). Patient recruitment started on February 11, 2019 and was completed on May 14, 2019. Data collection started on February 25, 2019 and was completed on February 28, 2020. We enrolled 82 participants. Data analysis is underway and we expect to publish results in January 2021.

## Discussion

HIART may have substantial benefit, including improving body composition, strength, and fitness, in middle-aged adults with low lean soft tissue mass. HIART is a time-efficient form of exercise, enabling busy middle-aged adults to meet physical activity recommendations while maximizing training results.

This RCT is rigorously designed, allowing conclusions to be formed about the acceptability and effectiveness of a supervised, group-based HIART intervention in middle-aged adults with low lean soft tissue mass. The findings can inform the development of future prevention-focused interventions aimed at counteracting the high prevalence of sarcopenia in the aging population.

## Abbreviations

ALMI: appendicular lean soft tissue mass index
DXA: dual energy X-ray absorptiometry
ECG: electrocardiogram
ESES: Exercise Self-Efficacy Scale
HIART: high-intensity interval aerobic resistance training
HIIT: high-intensity interval training
HR: heart rate
LAQ: Lifestyle Appraisal Questionnaire
PACES: Physical Activity Enjoyment Scale
RCT: randomized controlled trial
SF-12: 12-item Short Form Health Survey
O_2_max: maximum oxygen consumption
